# Interleukin-12p40 Modulates Human Metapneumovirus-Induced Pulmonary Disease in an Acute Mouse Model of Infection

**DOI:** 10.1371/journal.pone.0037173

**Published:** 2012-05-14

**Authors:** Krishnendu Chakraborty, Zehua Zhou, Nobuko Wakamatsu, Antonieta Guerrero-Plata

**Affiliations:** 1 Department of Pathobiological Sciences, Louisiana State University, Baton Rouge, Louisiana, United States of America; 2 Center for Experimental Infectious Disease Research, Louisiana State University, Baton Rouge, Louisiana, United States of America; Louisiana State University Health Sciences Center, United States of America

## Abstract

The mechanisms that regulate the host immune response induced by human metapneumovirus (hMPV), a newly-recognized member of the *Paramyxoviridae* family, are largely unknown. Cytokines play an important role in modulating inflammatory responses during viral infections. IL-12p40, a known important mediator in limiting lung inflammation, is induced by hMPV and its production is sustained after the resolution phase of infection suggesting that this cytokine plays a role in the immune response against hMPV. In this work, we demonstrated that in mice deficient in IL-12p40, hMPV infection induced an exacerbated pulmonary inflammatory response and mucus production, altered cytokine response, and decreased lung function. However, hMPV infection in these mice does not have an effect on viral replication. These results identify an important regulatory role of IL-12p40 in hMPV infection.

## Introduction

Human metapneumovirus (hMPV) is an emerging RNA virus of the *Paramyxoviridae* family that causes clinical syndromes ranging from mild illnesses, such as common cold, to more devastating conditions, such as bronchiolitis and pneumonia, [1], [2] similar to that seen in respiratory syncytial virus (RSV) infection. Identification and isolation of the virus was achieved in 2001 from nasopharyngeal aspirates of young children with respiratory disease [3] and it is currently recognized as a global and significant respiratory pathogen. Serological evidence indicate that hMPV seropositivity is almost universal by 5 years of age [3], [4] and by the age of 25, all adults have been infected with this virus [5]. The nosocomial impact of hMPV is estimated to be as high as that for RSV. hMPV is responsible for 5 to 15% of hospitalizations of children suffering from acute respiratory tract infections [6], [7], [8]. Elderly patients are also a target population for hMPV infection since outbreaks of hMPV have been reported in long term care facilities with high mortality rate in frail elderly residents [9], [10], [11], [12].

The immune response triggered by respiratory viruses plays a major role in the resolution of the virus-induced disease and host protection. Despite the significant progress made in the last ten years toward understanding the nature of hMPV infection [13], there remain significant questions regarding the immune response induced by this virus. hMPV is able to induce the production of several cytokines and chemokines in the human [14] and mouse [15] respiratory tract. Likely such mediators play crucial roles in mediating recovery from the viral infection. Therefore, the regulation of the immune response by specific cytokines in hMPV infection warrants further investigation. IL-12p40 is a regulatory cytokine that is induced and distinctly sustained during the course of hMPV infection [15], but the role of this cytokine in the context of the pathogenesis induced by hMPV, is currently unclear. IL-12p40 is a component of IL-12p70 and was shown to associate not only with the IL-12 p35 chain, but also with a p19 chain to form a covalently linked heterodimeric cytokine, IL-23 [16]. IL-12p40 can also be secreted as a monomer (IL-12p40) and as a homodimer (IL-12p80) [17]. IL-12p40 is often secreted in large excess over p70 heterodimer, p35 is only secreted as a part of the heterodimer when p40 is also produced by the same cells [16]. IL-12p40 is produced primarily by activated macrophages, dendritic cells (DC), neutrophils and microglia [16], [18], but it also produced by keratinocytes [19] and airway epithelial cells [20], [21]. Different functions have been attributed to IL-12p40, initially as an antagonist of IL-12p70 activity [17], [22], [23]. However, in contrast to this negative regulatory role, it has been reported that this cytokine plays a pivotal agonistic role in initiating the immune response [24] and performs several immunostimulatory functions like DC migration [25], macrophage inflammation [26], [27], and to trigger IFN-γ production by NK [28] and T cells [16]. Furthermore, several reports have provided convincing evidence indicating a regulatory role of IL-12p40 in pulmonary inflammation and host immune responses in experimental models of asthma [29] and infectious diseases [30], [31], [32]. However, the role that IL-12p40 plays in hMPV infection, has not yet been elucidated.

In the present study, we investigated the role of IL-12p40 in the inflammatory and immune responses in an experimentally induced hMPV infection by using IL-12p40-deficient mice. Our results indicate that IL-12p40 plays a major role in limiting hMPV disease by controlling body weight loss, reducing lung inflammation and airway mucus production and regulating pulmonary cell infiltration and cytokine release. However, the absence of IL-12p40 did not have a major impact in lung viral replication. These studies indicate a significant contribution of IL-12p40 to the protective host response to hMPV.

## Results

### IL-12p40 production in hMPV infection is sustained and its absence exacerbates disease severity

**Figure 1:**
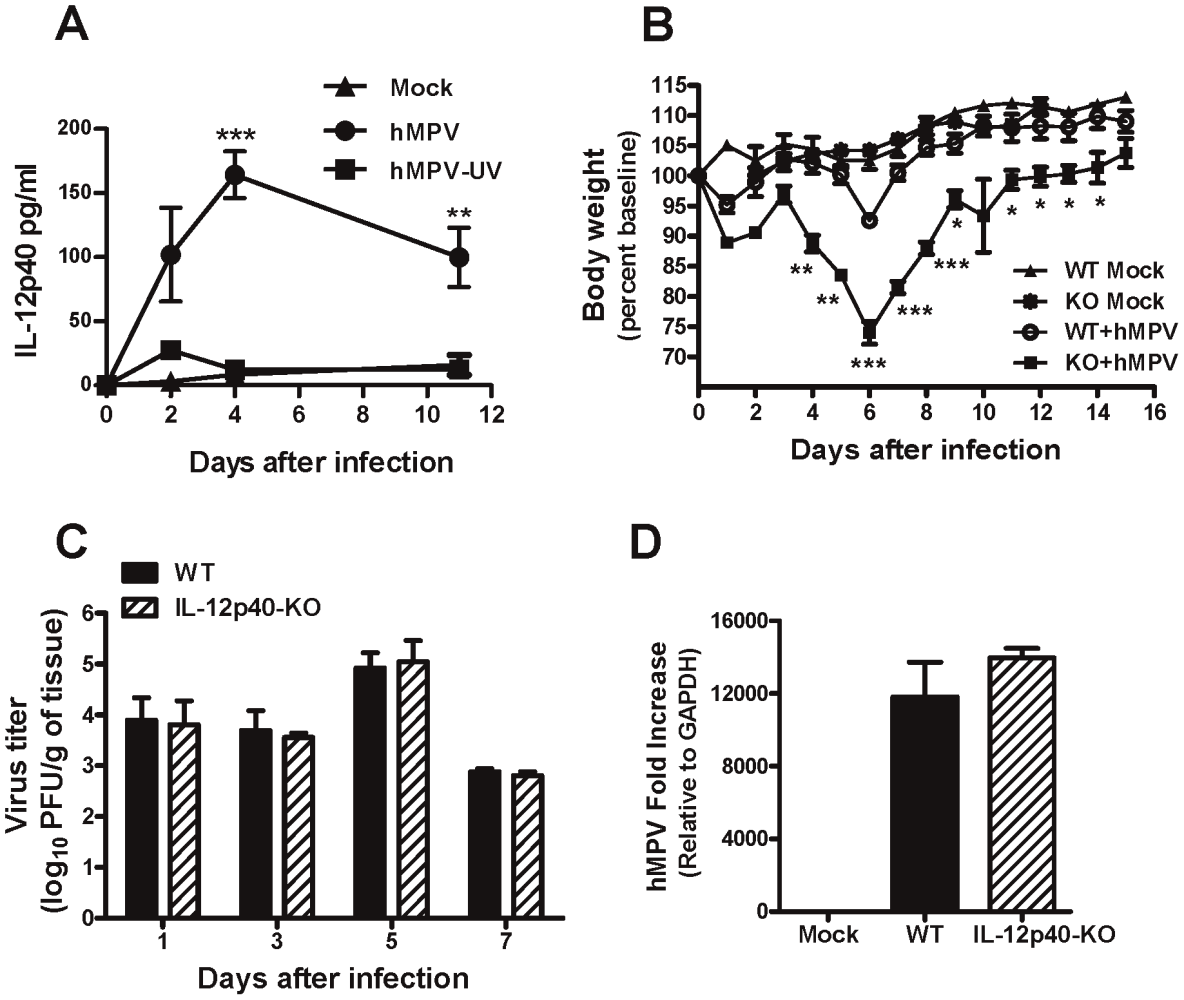
Disease severity and lung viral replication in the absence of IL-12p40. Mice (7–10 week old) were inoculated with PBS (mock) or infected i.n. with 5×10^6^ PFU of hMPV. (A) Bronchoalveolar lavage was collected at different time points from mock, hMPV-infected and UV-inactivated hMPV-infected mice and IL-12p40 was determined by Bio-Plex Pro™ assay. n = 5 mice/group. (B) Mice were monitored daily and body weight was calculated based on the original weight before the infection. n = 3 mice/group. (C) Total lung tissue was harvested at different time points and lung infectious viral particles were titrated on LLC-MK2 cell monolayers by methylcellulose plaque assay. n = 4 mice/group. (D) hMPV N gene expression in the lung of infected mice was determined by real-time RT-PCR. n = 3 mice/group. *P<0.05, **P<0.01, ***P<0.001.

In order to determine the role of IL-12p40 in the regulation of disease severity during hMPV infection, the production of IL-12p40, body weight loss, and lung viral replication were assessed. WT C57BL/6 and IL-12p40-KO mice were infected intranasally ( i.n.) with hMPV (5×10^6^ PFU/mouse), monitored daily and bronchoalveolar lavage (BAL) fluid was analyzed at different time points after infection. As shown in Fig. 1A, hMPV induced the release of IL-12p40 in BAL samples of C57BL/6 mice as early as day 1 after infection and the production of this cytokine remained sustained beyond the resolution of the infection (day 11, latest time point tested). Moreover, the induction of IL-12p40 was dependent of viral replication since UV inactivation of the virus resulted in a significant reduction of the production of the cytokine (Fig. 1A). In addition, the absence of IL-12p40 exacerbated the disease severity of hMPV-infected mice. As shown in Fig. 1B, IL-12p40-KO mice exhibited more body weight loss (26%) than WT mice (8%) at the peak of the clinical disease at day 6. Furthermore, a delayed recovery to baseline weight in IL-12p40-KO mice was also observed since those mice reached similar values to mock-infected mice at day 15 after infection versus at day 10 in the WT group (Fig. 1B). To determine whether the exacerbation of body weight loss was related to an altered viral replication in the deficient mice, hMPV-infected IL-12p40-KO and WT mice were sacrificed at day 1, 3, 5, and 7 after infection and total lung tissue was collected to determine virus replication by plaque assay. Resulting viral titers showed that hMPV replicates in the lungs of WT and IL-12p40-KO mice with a peak of viral titer at day 5 after infection. However, no difference between both groups of animals was observed in any time point tested (Fig. 1C). Differences in viral replication were confirmed by the analysis of viral gene expression in the lungs at day 5 after infection (peak of viral replication), where the virus was detected in the infected animals, but in similar amounts when compared WT and IL-2p40-KO mice (Fig. 1D).

### Lack of IL-12p40 exacerbates pulmonary inflammation in hMPV-infected mice

**Figure 2:**
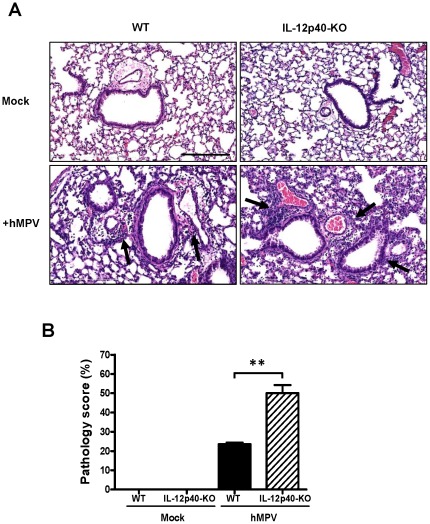
Pulmonary inflammation in hMPV-infected IL-12p40-KO mice. C57BL/6 and IL-12p40-KO mice were infected i.n. with 5×10^6^ PFU of hMPV or mock-treated and lungs were harvested at day 7 after hMPV infection, fixed for slide preparation and H&E stained. (A) Representative stained lung tissue sections from the indicated treatment. Arrows indicate cells infiltrating the perivascular and peribronchial spaces, Scale bar = 200 µm. (B) Pathology score of prepared slides (scored as described in Materials and Methods). n = 5 mice/group. **P<0.01.

Next, we determined the role of IL-12p40 in the regulation of lung inflammation in response to hMPV infection. Pathology score and differential cell count in BAL fluid were assessed in WT and IL-12p40-KO mice. Groups of animals were infected i.n. with 5×10^6^ PFU of hMPV and lung tissue and BAL fluid were harvested at day 7 after infection (when pulmonary inflammation peaks [33]). Lung sections were processed and stained by Hematoxylin and eosin (H&E) and cells from BAL samples were counted, cytospun and analyzed for differential cell analysis. Lung histopathology analysis demonstrated that no airway inflammation was observed in mock-infected WT or KO mice (Fig. 2A, upper panels). However, hMPV infection induced a cellular infiltration in the perivascular and peribronchial spaces of WT mice (Fig. 2A, lower left panel) which was exacerbated in the absence of IL-12p40 (Fig. 2A, lower right panel). Quantification of the pulmonary inflammation, represented as pathology score, indicated that the inflammation in the respiratory tract of IL-12p40-KO mice was increased over two-fold when compared to WT animals (Fig. 2B).

**Figure 3:**
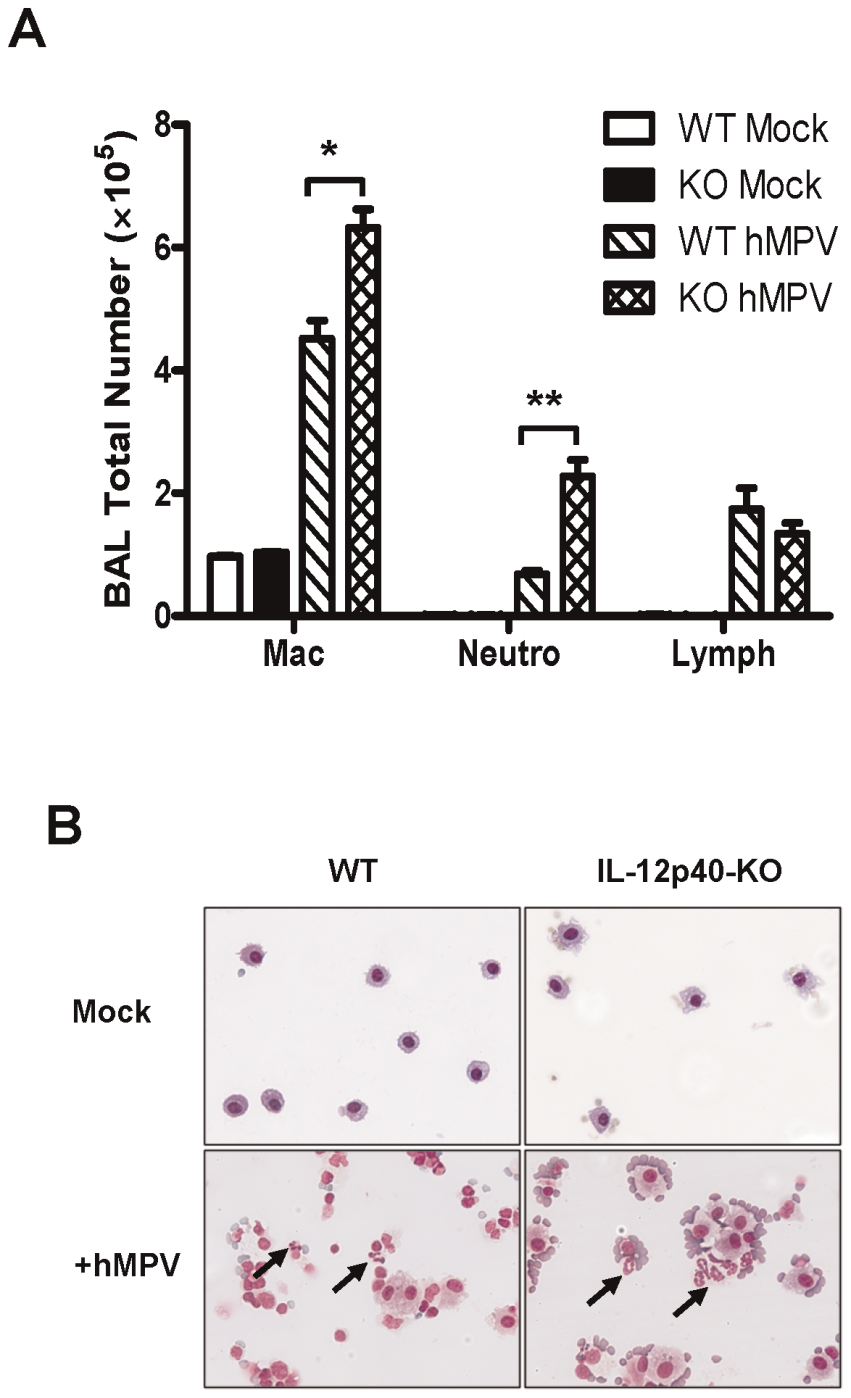
Total differential cell counts in hMPV-infected IL-12p40-KO mice. C57BL/6 and IL-12p40-KO mice were infected i.n. with 5×10^6^ PFU of hMPV and BAL samples were harvested at day 7 after hMPV infection. Cell preparations were stained (Wright-Giemsa) and counted under the microscope (200 cells/slide). Bar graph represents mean ± SEM. n = 9 mice/group. *P<0.05, **P<0.01. (B) Representative stained cytospin preparations from the indicated treatment. Arrows indicate neutrophils infiltrating the alveolar spaces.

The effect of IL-12p40 on pulmonary inflammation was confirmed by the analysis of the cell populations in BAL samples. As shown in Fig. 3A, the total number of macrophages, neutrophils, and lymphocytes was increased after hMPV infection. Moreover, in the absence of IL-12p40, the number of macrophages and neutrophils was significantly increased in the BAL samples from IL-12p40-KO mice infected with hMPV as compared with that of WT hMPV-infected. In particular, an exacerbated increase in BAL neutrophils was noted in IL-12p40-KO (2.3×10^5^±0.2) vs WT (0.7×10^5^±0.05) hMPV-infected mice. Numbers of lymphocytes were increased after hMPV infection, but no significant difference was observed when WT and KO groups of mice were compared. No eosinophils were observed in the BAL samples of any of the groups of mice analyzed. Alveolar macrophages were mostly recovered from samples of mock-infected mice in both groups of animals (Fig. 3B, upper panels). After hMPV infection, an increased frequency of lymphocytes and neutrophils was observed. As shown in Fig. 3B (lower panels), the frequency of neutrophils in the alveolar spaces was further exacerbated in the absence of IL-12p40.

### Increased goblet cell formation and mucin gene expression in the airways of IL-12p40-KO mice following hMPV infection

**Figure 4:**
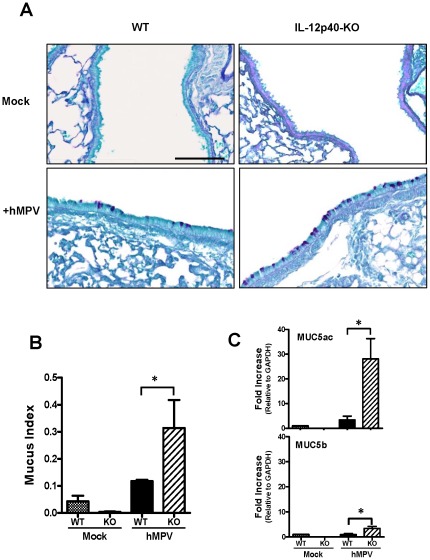
Mucus production after hMPV infection in IL-12p40 deficient mice. C57BL/6 and IL-12p40-KO mice were infected i.n. with 5×10^6^ PFU of hMPV or mock-infected and lungs were harvested at day 7 after hMPV infection. (A) Lung tissue was fixed for slide preparation, PAS stained and tissue sections were digitalized. Representative stained lung tissue sections from the indicated treatment. Mucus-producing cells in the airways were identified by positive PAS staining. Scale bar = 100 µm. (B) Each area of airway epithelium was measured to calculate the Mucus index (as described in Materials and Methods). An average of 52 individual airways were measured per mouse. n = 3 mice/group. (C) RNA was isolated from the lungs of mock- and hMPV-infected mice (WT and KO) and transcribed into cDNA. Samples were assessed for expression of MUC5ac and MUC5b using quantitative RT-PCR by SYBR green. Each sample was normalized using GAPDH control and the bar graphs represent average fold increase to RNA obtained before infection. n = 3 mice/group. *P<0.05.

Increased production of mucus in the airways is usually correlated with exacerbated lung inflammation. In order to determine whether the absence of IL-12p40 would also alter the mucus production, the presence of mucin-expressing goblet cells in the airways as well as the expression of mucins in lung, were investigated. Lung sections from mice infected i.n. with 5×10^6^ PFU of hMPV, were collected at day 7 after infection [same time point as in the determination of pulmonary inflammation, (Fig. 2)]. Periodic acid-Schiff (PAS) staining was used to detect mucin-expressing cells in the airway epithelium. In mock-infected WT and IL-12p40-KO mice, the presence of goblet cells was almost absent (Fig. 4A, upper panels). However, after hMPV infection, the number of goblet cells was increased in the airways of WT mice (Fig. 4A, left lower panel) and further exacerbated in those mice lacking the expression of IL-12p40 (Fig. 4A, right lower panel). Quantification of mucus producing cells in the airways was determined by assessing the mucus index (as described in Methods). As shown in Fig. 4B, the mucus index was increased after hMPV infection in WT mice. Furthermore, we observed an increased mucus index in the absence of IL-12p40 by more than two times when compared to WT mice infected with hMPV. In order to confirm the exacerbated mucus production as indicated by the mucus index, in a separate set of experiments, the expression of mucin 5 subtype AC (Muc5ac) and Muc5b was investigated. Muc5ac and Muc5b have been implicated as markers of goblet cell metaplasia in lung pathologies based upon expression studies in humans, in animal models, and in cell cultures [34], [35], [36]. Therefore, lung tissue was collected at day 7 after hMPV infection to quantify the expression of the mucins by quantitative RT-PCR. As shown in Fig. 4C, the expression of Muc5ac was modestly induced after hMPV infection in WT mice when compared to WT mock- infected mice. However, following hMPV infection, the absence of IL-12p40 exacerbated Muc5ac expression over 8 times when compared to WT mice. On the other hand, Muc5b was not induced after hMPV infection in WT mice, as similar expression was observed in mock-infected mice. However, it was increased in IL-12p40-KO mice after hMPV infection, although in less magnitude than Muc5ac. Thus, the absence of IL-12p40 resulted in significantly increased mucin expression in the airways of hMPV-infected mice.

### IL-12p40 expression regulates the production of IFN-γ, IL-6, CXCL10, CCL11, CXCL1 and CCL2 in mice infected with hMPV

**Figure 5:**
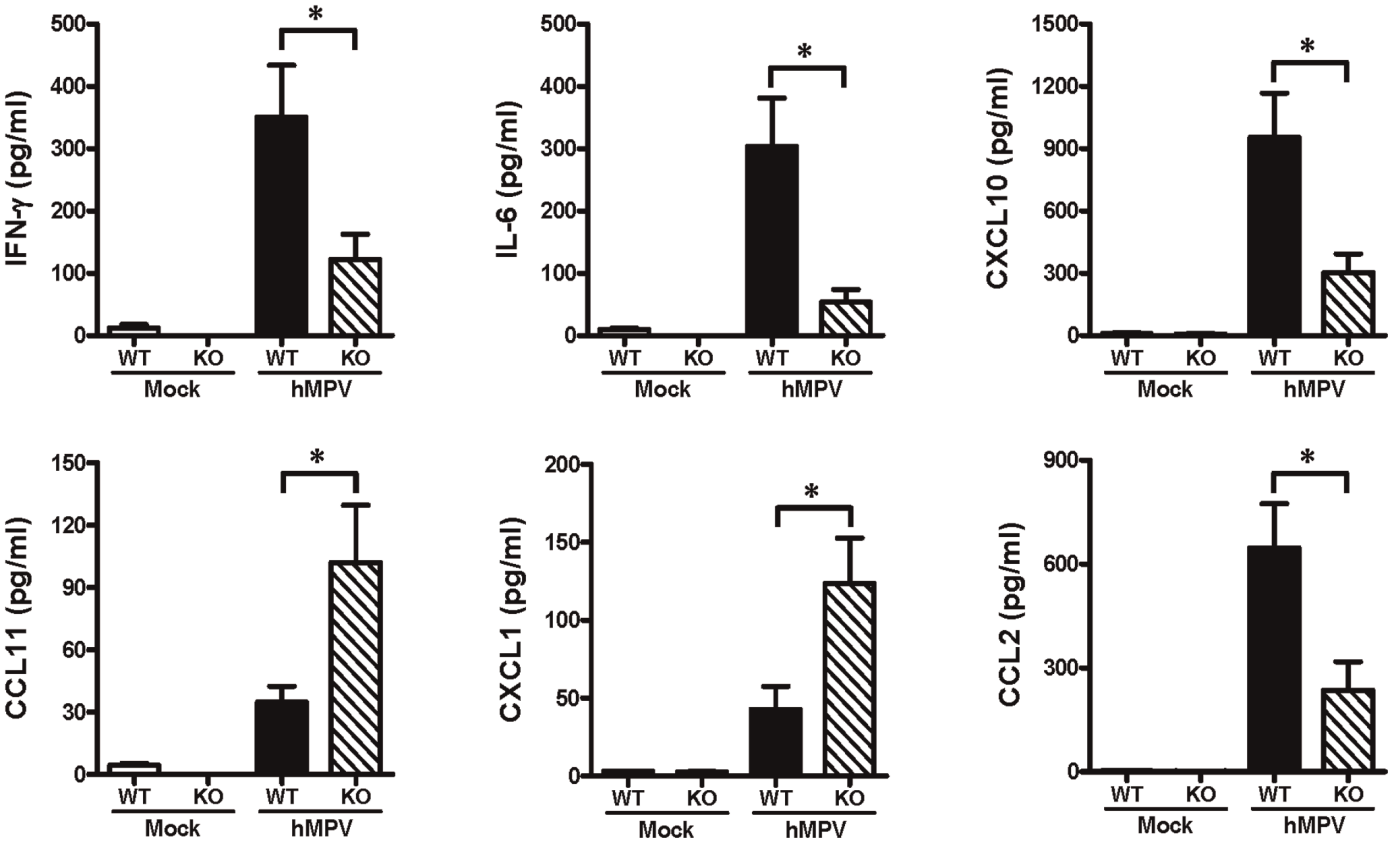
Cytokine and chemokine production in the lung of IL-12p40-KO mice infected with hMPV. WT and IL-12p40-KO mice were infected i.n. with 5×10^6^ PFU of hMPV or mock-infected and sacrificed at day 7 after infection. BAL samples were collected from each group of mice and assessed for cytokine/chemokine production by a multi-Plex Cytokine detection system. n = 7–8 mice/group.*P<0.05.

To define the role of IL-12p40 in the regulation of hMPV-induced cytokine response, the level of cytokines and chemokines was assessed in IL-12p40-KO mice and compared to WT ones. Mice were infected i.n. with hMPV, and at day 7 after infection, BAL samples were collected from each group of mice and assessed for the presence of cytokines by using a multi-plex cytokine detection system. Our data indicate that, compared to immunocompetent mice, the lack of IL-12p40 significantly decreased the production of the Th1 regulatory cytokine IFN-γ and the proinflammatory cytokine IL-6 by 65% and 82%, respectively (Fig. 5). A similar effect was observed with the release of the chemokines CXCL10 (IP-10) and CCL2 (MCP-1), where their production was decreased by over 60% in both cases. On the other hand, the production of CCL11 (Eotaxin) and CXCL1 (KC, IL-8 homologue), cytokines that regulates the chemotaxis and function of neutrophils and eosinophils, was upregulated in the absence of IL-12p40. Expression of CCL11 and CXCL1 was increased over two times in the deficient mice in response to hMPV infection when compared to WT mice. Finally, production of CXCL2 (MIP-2), CCL4 (MIP-1β), CCL3 (MIP-1α), and CXCL9 (MIG) was induced after hMPV infection but no changes were observed in IL-12p40-KO vs WT mice (not shown). Overall, these data indicate that IL-12p40 regulates the cytokine response in hMPV infected mice.

### Increased decline in lung function in IL-12p40-KO mice infected with hMPV

**Figure 6:**
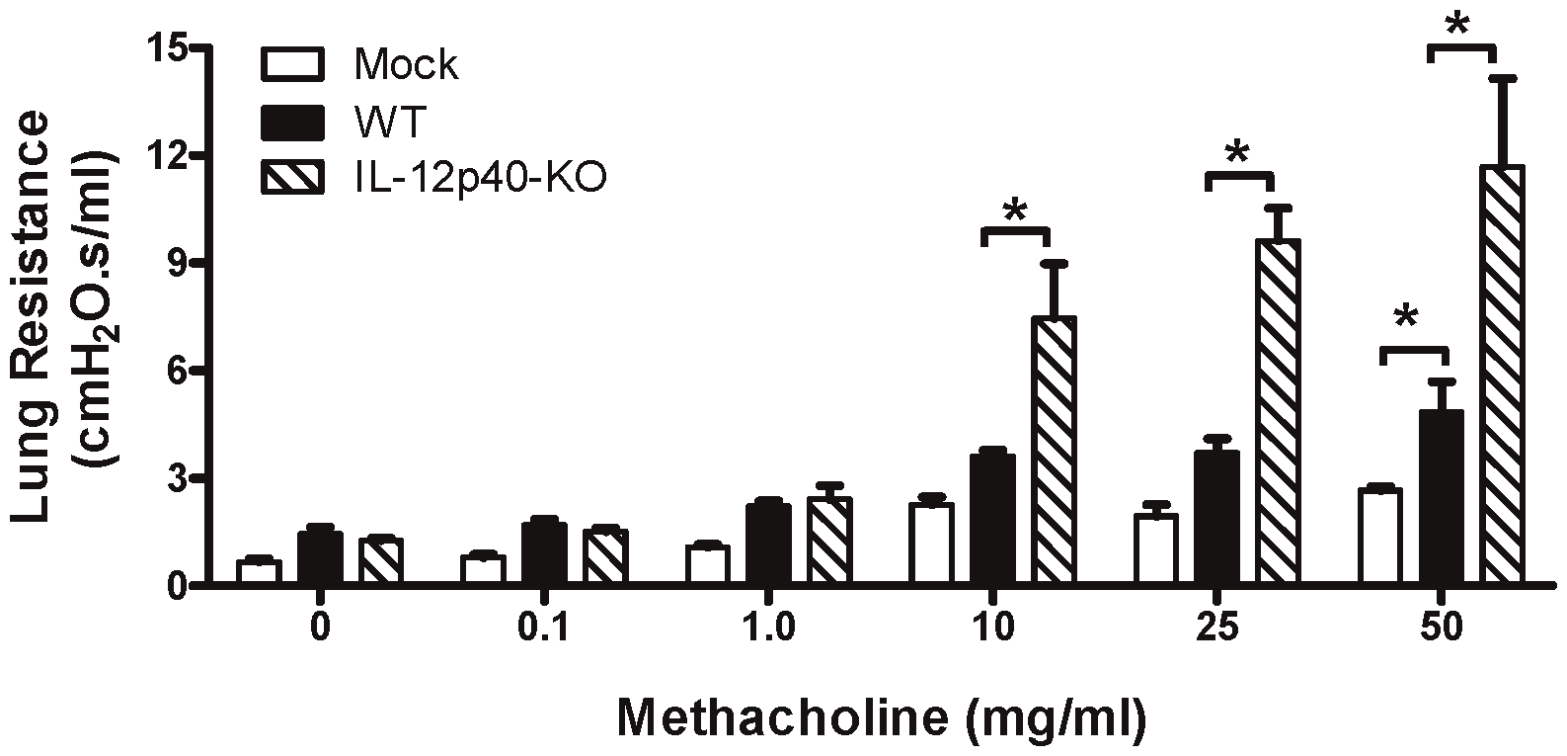
Lung function in IL-12p40-KO mice infected with hMPV. WT or IL-12p40-KO mice were infected with hMPV (5×10^6^ PFU/mice), and airway resistance was measured at day 15 after hMPV infection. Change in airway resistance between baseline values after challenge with different concentrations of aerosolized methacholine (0.1, 1, 10, 25, and 50 mg/ml) was measured by the Flexivent system (Resistance units = cmH_2_O.s/mL). Values represent mean ± SEM. n = 4 mice/group. *P<0.05.

To assess the role of IL-12p40 on pulmonary function after hMPV infection, WT and IL-12p40-KO mice were infected with hMPV (or mock infected as baseline control) and then invasive assessment of lung function 15 days later was conducted. No differences in lung resistance were observed between the WT and IL-12p40-KO groups at concentrations of methacholine of 0.1 and 1.0. However, administration of higher concentrations of methacholine revealed significant functional changes in IL-12p40-KO mice. When compared to WT, deficient animals showed over a two-fold increase of lung resistance at doses of 10, 25, and 50 mg/ml of methacholine. That difference was increased one more fold when compared to mock-infected groups (Fig. 6).

## Discussion

Human metapneumovirus induces a sustained production of IL-12p40 in the lung of BALB/c [15] and C57BL/6 mice (Fig. 1A), suggesting an important role in hMPV infection. In order to determine the role of IL-12p40 in hMPV-induced pathogenesis, we used an IL-12p40-KO mouse model of infection. A series of parameters including body weight loss, pulmonary inflammation and mucus production, viral replication, production of cytokines, cellular infiltration in the lung, and lung function were determined to assess hMPV pathogenesis. The present study shows that the absence of IL-12p40 in hMPV-infected mice induced an exacerbated inflammatory response as well as increased numbers of goblet cells and body weight loss. Those findings go in line with the reduced lung function observed in this model. Cytokine secretion was also perturbed by the lack of IL-12p40, but not the viral replication in the lung. These data indicate that IL-12p40 plays an important regulatory role in the pathogenesis induced by hMPV infection.

The production of IL-12p40 was detected in BAL samples of hMPV-infected C57BL/6 mice. Therefore, previous data reporting the production of IL-12p40 in BALB/c mice [15] were reproduced in this work. We also observed that the induction of the cytokine was dependent of viral replication as demonstrated by the reduced amounts of IL-12p40 after the UV-inactivation of the inoculum. Body weight loss is a parameter to monitor the severity of the disease after hMPV infection [37]. In this study, the body weight loss was increased in IL-12p40-KO mice, indicating that IL-12p40 has a protective role against hMPV-induced illness. Studies in BALB/c mice infected with RSV have shown, however, that IL-12p40 does not influence weight loss in the infected mice, suggesting that IL-12p40 distinctly regulates the host response induced by these two paramyxovirus infections [38]. On the other hand, the observed exacerbated disease in IL-12p40-KO mice infected with hMPV does not seem to be dependent on viral replication since no differences in lung viral titer or viral gene expression were found in the absence or presence of IL-12p40. This finding indicates that IL-12 is not essential to control lung viral clearance. These data are consistent with studies reported in RSV infection, where the lack of IL-12p40 did not alter the viral replication in infected mice [31].

Pulmonary inflammation represents a critical host response to control viral infections in the lung. However, an exacerbated inflammatory response may result in a severe lung disease. In this work, we showed that airway inflammation was increased in IL-12p40-KO mice as compared with that of WT mice infected with hMPV. Moreover, increased number of macrophages and neutrophils was found in BAL samples of IL-12p40-KO mice, consistent with the increased pathology score observed during hMPV infection. Similar to the findings reported in the current work, studies in an experimental model of RSV infection indicate that the lack of IL-12p40 also exacerbated the recruitment of inflammatory cells including macrophages and neutrophils at day 7 after infection [31], [38]. In addition, treatment of RSV-infected mice with anti-IL-12 polyclonal antibodies also resulted in an exacerbated lung inflammatory response at day 8 and 12 after infection [39]. However, the data reported here is the first evidence demonstrating that IL-12p40 plays a critical role in controlling pulmonary inflammation during hMPV infection. Furthermore, the observed exacerbated inflammatory response was accompanied by a goblet cell hyperplasia and mucus hypersecretion in IL-12p40-KO mice following hMPV infection. Similar to our findings, the lack of IL-12p40 in a mouse model of RSV infection increased the number of mucus-secreting cells [31]. Globlet cells are interspersed, and secrete mucins, which are high molecular mass, highly glycosylated macromolecules that are the major components of mucus secretions [40]. Mucus overproduction is common in pulmonary conditions such as bronchiolitis and has been reported in mice infected with RSV and hMPV [31], [41], [42]. The importance of mucins as defensive molecules in adult airways is suggested by their abundance during homeostasis and their up-regulation during inflammation. In this work, we observed that only Muc5ac was induced after hMPV infection, but not Muc5b. However, both mucins were upregulated in the absence of IL-12p40, predominantly Muc5ac. Muc5b has been identified as the predominant secreted gel-forming mucin expressed in healthy adult murine lungs [43], [44], whereas Muc5ac is the most predominantly induced gel-forming mucin in antigen challenged murine lungs [43] and the one expressed by human airway epithelial cells [45]. Overall, our data suggest that Muc5ac is predominantly induced in the lungs of hMPV-infected mice and its production is regulated by the expression of IL-12p40.

Production of several inflammatory mediators is induced after hMPV infection [14], [15]. IL-12 has pleiotropic effects on NK and T cells and induces CD4+ T cells to assume a Th1 phenotype [16]. In the present study, the absence of IL-12p40 induced a decreased production of the Th1-derived cytokine IFN-γ, IL-6, CCL2, and CXCL10 and exacerbated the expression of CCL11 and CXCL1. IFN-γ is a critical cytokine for host defense against viral infections, as it activates macrophages to kill intracellular microbes [46]. In support of our observations, IFN-γ production has been reported to be downregulated in mice lacking IL-12p40 after infection with *Mycobacterium tuberculosis* [32]. However, different to our work, in IL-12p40-KO mice infected with RSV, the production of IFN-γ remained unchanged [31]. This discrepancy could be attributable to the different experimental conditions. In particular, the different viral pathogens and the distinct genetic background of the IL-12p40-KO mice used in each experimental model. IFN-γ can influence the production of other cytokines including IL-6, CXCL10, CCL11, and CXCL1. IL-6 is a multifunctional cytokine involved in the control of many cell functions, including the activation of the immune system, stem cell differentiation, the maintenance of bone homeostasis, and in many neural functions [47]. Several reports indicate that IFN-γ enhances IL-6 gene expression and IL-6 production in monocytes [48], [49]. It remains probable, therefore, that the reduced production of IL-6 observed in IL-12p40-KO mice infected with hMPV, might be a consequence of the observed decreased production of IFN-γ. The finding that IL-6 induces neutrophil apoptosis [50] supports the notion that IL-6 substantially contributes to the resolution of acute neutrophil infiltration. However, whether the reduced production of IL-6 in IL-12p40-KO mice was related to the increased numbers of neutrophils found in the BAL samples, remains to be investigated. CXCL10 (IFN-γ-inducible protein 10, IP-10) production is directly regulated by IFN-γ [51] and its decreased production in the IL-12p40-KO mice may be directly related to the lower production of IFN-γ.

On the other hand, IFN-γ has an inhibitory activity on the expression and secretion of IL-8 (CXCL1) [52], [53], [54], suggesting that the downregulation of IFN-γ production in mice lacking IL-12p40 may have an impact on the production of CXCL1 in hMPV-infected mice. IL-8 is a potent neutrophil chemotactic and activating cytokine. Moreover, regulated recruitment and clearance of neutrophils is the hallmark of competent host defense and resolution of inflammation. Neutrophil recruitment after hMPV infection has been observed up to day 7 (Fig. 3A) or up to day 14 [55]. In this work, the recruitment of neutrophils was increased by the lack of IL-12p40. Therefore, although not addressed in this work, there is the possibility that the observed increased production of CXCL1 in hMPV-infected mice in the absence of IL-12p40, can be correlated with the increased recruitment of neutrophils in the same animals. IFN-γ can also regulate the production of CCL11. Studies in vitro indicate that IFN-γ is able to inhibit the production of CCL11 in fibroblasts [56]. However, despite the increased amount of CCL11 in the absence of IL-12p40, no eosinophils were found in hMPV-infected IL-12p40-KO mice.

Previous studies have shown that lung function is decreased after hMPV infection [55]. Based on the present observations, we demonstrate that lung function in infected mice is regulated by IL-12p40 as lung resistance was exacerbated in infected mice in the absence of IL-12p40 (Fig. 6). This finding is in line with those from RSV-infected mice and treated with anti-IL-12 antibodies [39] and with the increased lung resistance of IL-12p40-KO mice after acute or prolonged exposure to allergen [29]. Taken together, these data indicate that IL-12 may play a key role in regulating lung function during hMPV infection.

In conclusion, our studies demonstrate that IL-12p40 is critically involved in the processes of lung inflammation, cytokine release and airway hyperresponsiveness in mice experimentally infected with hMPV. The results presented herein suggest that IL-12p40 plays a central role in protection against hMPV infection and support other indirect evidence of the involvement of lung cytokines in the clinical manifestations of naturally acquired hMPV disease. However, further studies analyzing the specific production of IL-12p40 in the respiratory tract of hMPV-infected individuals are needed. Determining the significance of the role of IL-12p40 in immunity is important for our understanding of the factors required for initiating the immune responses to hMPV infection.

## Methods

### Virus stocks

hMPV (strain CAN97-83) initial stocks were provided by the Respiratory Virus Section, Centers for Disease Control (CDC), Atlanta, GA, with permission from Dr. Guy Boivin at the Research Center in Infectious Diseases, Regional Virology Laboratory, Laval University, Quebec City, Canada. Virus was propagated and titrated in LLC-MK2 cells (ATCC, Manassas, VA) in the presence of trypsin (Worthington, Lakewood NJ), as described elsewhere [33], [57].

### Ethic statement

Animal care and use were conducted in accordance with the National Institutes of Health and Louisiana State University institutional guidelines. The Louisiana State University Animal Care and Use Committee specifically approved this study under the protocol number: 09-050. Mice were housed in a temperature-controlled room with proper darkness-light cycles, fed with a regular diet, and maintained under the care of the Division of Laboratory Animal Medicine facility, Louisiana State University, Baton Rouge, Louisiana. The mice were sacrificed by an intraperitoneal injection of ketamine and xylazine and exsanguinated via the femoral vessels.

### Mice and infection protocol

Female and male B6.129S1-*Il12b^tm1Jm^*/J (IL-12p40-KO) and C57BL/6J WT were obtained from The Jackson Laboratory (Bar Harbor, ME). All mice were bred in specific pathogen free conditions and used when 7 to 10 weeks old. Under light anesthesia, mice were infected i.n. with 50 µl of hMPV diluted in PBS (final administered dose: 5×10^6^ PFU). In some experiments mice were inoculated with inactivated virus. Virus stocks were exposed for 10 minutes to UV irradiation using ultraviolet lamp (254 nm wavelength). After this treatment, no infectious virus was detected in the inoculum used when tested by plaque assay (not shown). As mock treatment, mice were inoculated with an equivalent volume of PBS (herein referred as mock).

### Broncholaveolar lavage

To collect a bronchoalveolar lavage (BAL), the lungs were flushed twice with ice-cold sterile PBS (1 ml). A total of 100 µl of this BAL fluid from each mouse was retained for cytospin analysis, and the rest was immediately centrifuged and stored at −70°C until analysis. BAL differential cell counts were determined using morphogenic criteria under light microscopy of Wright-Giemsa-stained cytospin with a total count of 200 cells per slide. Total number of BAL cells was counted by trypan blue.

### Detection of cytokines and chemokines

Levels of cytokines and chemokines in BAL fluid were determined with the Milliplex MAP ™ 32-Mouse Plex Cytokine detection system (Millipore, Billerica, MA) or Bio-Plex Pro™ assay (Bio-Rad, Hercules, CA), according to the manufacturer's instructions. The range of the sensitivity of the assays is 3.2 to 10000 pg/ml.

### Pulmonary histopathology

For histological analysis, lungs were perfused and fixed in 10% buffered formalin and embedded in paraffin. Multiple 4 µm-thick sections were stained with haematoxylin & eosin (H&E) to assess lung inflammation or periodic acid Schiff (PAS) to identify mucus-secreting cells. A blind analysis and scoring for cellular infiltration was performed by a board certified pathologist, as previously described [58], [59]. Briefly, inflammatory infiltrates were scored by enumerating the layers of inflammatory cells surrounding the vessels and bronchioles. The number of abnormal perivascular and peribronchial spaces divided by the total perivascular and peribronchial spaces was the percentage reported as the pathology score. Mucus index was quantified in additional lung sections stained PAS to assess goblet cell hyperplasia. PAS stained slides were digitally scanned using the high throughput slide scanner NanoZoomer with a 20× objective and areas of airway epithelium were measured using the NanoZoomer Digital Pathology view software (both from Hamamatsu Photonics, Hamamatsu City, Japan). All airways present in the lung sections were analyzed. The mucus index was calculated as follows: the area of airway epithelium PAS positive/total area of the conducting airway epithelium.

### Lung viral replication

Lungs were removed from infected animals at days 1, 3, 5, and 7 after hMPV infection. Tissue samples were homogenized in 1 ml of Dulbecco's modified Eagle's medium and centrifuged twice at 10,000× g for 1 min at 4°C. Serial two-fold dilutions of the supernatant were determined by plaque assay on LLC-MK2 cells under methylcellulose overlay. Plaques were visualized 6 days later by HRP staining, as previously described [33], [57].

### Real time quantitative PCR

The first -strand cDNA was synthesized from RNA using the Maxima First strand cDNA Synthesis kit (Fermentas, Maryland MS) according to the manufacturer's instructions. cDNA fragments of interest were amplified using Maxima SYBR green/ROX qPCR Master Mix (Fermentas) or Faststart Universal probe master (Roche, Indianapolis, IN). For hMPV nucleoprotein (N) gene, predesigned Taqman assay primers and probe were used and MUC5b and MUC5ac, SYBR green assays primers were designed as mentioned elsewhere [60]. All assays for MUC5ac, MUC5b, hMPV, and GAPDH were run on the 7900HT Fast Real-Time PCR System following suggested manufacturer's cycling parameters (Applied Biosystems, Foster City, CA). The comparative CT (ΔΔCT) method was used to quantitate the expression of target genes which were normalized to endogenous reference (GAPDH) expression in reference to transcripts from uninfected and untreated control cells.

### Pulmonary function testing

Lung function was measured invasively on anesthetized mice using flexiVent® system (SCIREQ, Montreal, QC, Canada) including the heart rate monitor to ensure that appropriate anesthesia was maintained throughout the duration of ventilation. Airways hyperresponsiveness (AHR) to methacholine was assessed in mice infected for 15 d with hMPV as previously described [55]. AHR was measured after ultrasonic nebulization of 1× PBS (baseline) and in response to increasing doses of methacholine (0.1, 1.0, 10, 25, and 50 mg/ml; acetyl-β-methylcholine chloride, MP Biomedicals) delivered by ultrasonic nebulizer. Ventilation was maintained at a rate of 150 breaths/minute, a tidal volume of 7.5 ml/kg, and a positive end expiratory pressure of 3 cm of water. Mice were allowed to acclimate to the ventilator for 2–3 minutes before initiation of readings, and three to four total lung capacity functions were performed during this acclimation period to prevent atelectasis and to ensure maximum airway and alveolar recruitment. Peak responses during each 5-min period were determined for resistance. Respiratory mechanics were assessed using the linear first-order single compartment model, which provides resistance of the total respiratory system and the constant phase model, which utilizes forced oscillation to differentiate between airways resistance and peripheral tissue damping. Only measurements with a coefficient of determination of 0.95 or greater were used and measurements were repeated until a total of 3 pressure-volume curves and 3 single compartment perturbations, each with acceptable coefficients of determination, were obtained. The averages of these 3 measurements were determined for each mouse and averaged for each experimental group.

### Statistical analysis

Statistical significance was calculated by one-way ANOVA to ascertain differences between groups, followed by a Tukey-Kramer test to correct for multiple comparisons using Graph Pad InStat 3 (GraphPad Software, La Jolla, CA). Each experiment for the different parameters assessed was performed at least three times. Unless otherwise indicated, results are expressed as mean ± SEM.
